# 3-{1-[(2,4-Dinitrophenyl)hydrazino]­ethyl­idene}-5-(1-methylpropyl)pyrrolidine-2,4-dione

**DOI:** 10.1107/S1600536809012458

**Published:** 2009-04-08

**Authors:** David Siegel, Stefan Merkel, Matthias Koch, Franziska Emmerling, Irene Nehls

**Affiliations:** a Bundesanstalt für Materialforschung und -prüfung, Abteilung Analytische Chemie; Referenzmaterialien, Richard-Willstätter-Strasse 11, D-12489 Berlin-Adlershof, Germany

## Abstract

In the title compound, C_16_H_19_N_5_O_6_, two intramolecular N—H⋯O hydrogen bonds help to establish the conformation.  In the crystal, intermolecular N—H⋯O links result in chains propagating in [010].

## Related literature

For the use of the title compound in instrumental analytical chemistry, see: Siegel *et al.* (2009 ▶). For the crystal structure of the tenuazonic copper(II) salt, see: Dippenaar *et al.* (1977 ▶). For the structures of other 2,4-dinitro­phenyl­hydrazones, see: Tameem *et al.* (2006 ▶); Monfared *et al.* (2007 ▶); Valente *et al.* (2008 ▶); Yin *et al.* (2008 ▶). Solubilized tetra­mic acids and their hydrazones display a variety of tautomeric forms, see: Gelin *et al.* (1982 ▶); Nolte *et al.* (1980 ▶); Royles (1995 ▶); Yamaguchi *et al.* (1976*a* ▶, 1976*b* ▶). For the synthesis, see: Lebrun *et al.* (1988 ▶).
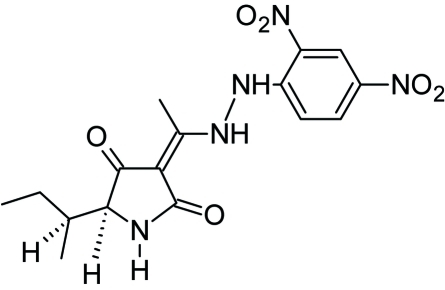


## Experimental

### 

#### Crystal data

C_16_H_19_N_5_O_6_
*M* *_r_* = 377.36
Monoclinic, 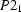

*a* = 10.6710 (10) Å
*b* = 4.9387 (5) Å
*c* = 16.839 (2) Å
β = 107.363 (4)°
*V* = 846.98 (15) Å^3^
*Z* = 2
Cu *K*α radiation
μ = 0.98 mm^−1^
*T* = 193 K
0.64 × 0.06 × 0.06 mm

#### Data collection

Enraf–Nonius CAD-4 diffractometer
Absorption correction: ψ scan (*CORINC*; Dräger & Gattow, 1971 ▶) *T*_min_ = 0.78, *T*_max_ = 0.94
3890 measured reflections
3282 independent reflections
3103 reflections with *I* > 2σ(*I*)
*R*_int_ = 0.025
3 standard reflections frequency: 60 min intensity decay: 2%

#### Refinement

*R*[*F* ^2^ > 2σ(*F* ^2^)] = 0.039
*wR*(*F* ^2^) = 0.111
*S* = 0.99
3282 reflections
247 parameters
1 restraint
H-atom parameters constrained
Δρ_max_ = 0.24 e Å^−3^
Δρ_min_ = −0.21 e Å^−3^
Absolute structure: (Flack,1983 ▶)
Flack parameter: 0.1 (2)

### 

Data collection: *CAD-4 Software* (Enraf–Nonius, 1989 ▶); cell refinement: *CAD-4 Software*; data reduction: *CORINC* (Dräger & Gattow, 1971 ▶); program(s) used to solve structure: *SIR97* (Altomare *et al*., 1999 ▶); program(s) used to refine structure: *SHELXL97* (Sheldrick, 2008 ▶); molecular graphics: *ORTEPIII* (Burnett & Johnson, 1996 ▶); software used to prepare material for publication: *SHELXTL* (Sheldrick, 2008 ▶).

## Supplementary Material

Crystal structure: contains datablocks I, global. DOI: 10.1107/S1600536809012458/kj2118sup1.cif

Structure factors: contains datablocks I. DOI: 10.1107/S1600536809012458/kj2118Isup2.hkl

Additional supplementary materials:  crystallographic information; 3D view; checkCIF report

## Figures and Tables

**Table 1:** Hydrogen-bond geometry (Å, °)

*D*—H⋯*A*	*D*—H	H⋯*A*	*D*⋯*A*	*D*—H⋯*A*
N3—H3*N*⋯O2	0.93	1.99	2.630 (2)	125
N4—H4*N*⋯O5	0.89	2.00	2.710 (2)	135
N3—H3*N*⋯O5^i^	0.93	2.36	2.949 (2)	121
N4—H4*N*⋯O5^i^	0.89	2.43	2.898 (2)	113
N5—H5*N*⋯O2^ii^	0.86	2.48	3.293 (2)	159

Symmetry codes: (i) 
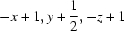
; (ii) 
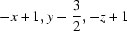
.

**Fig. 1.**
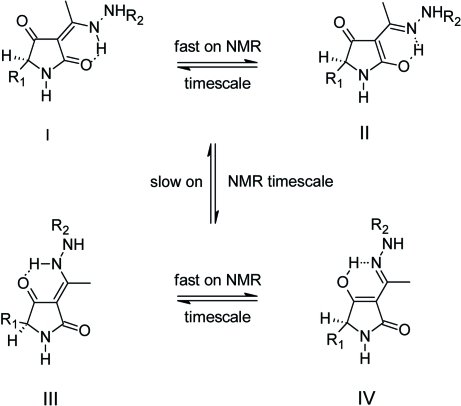
Tautomeric equilibria of tetramic acid dinitrophenylhydrazones.

**Fig. 2.**
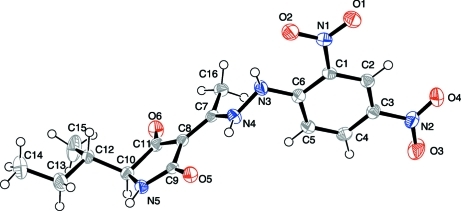
ORTEP representation of the title compound with atomic labeling, shown with 50% probability displacement ellipsoids.

**Fig. 3.**
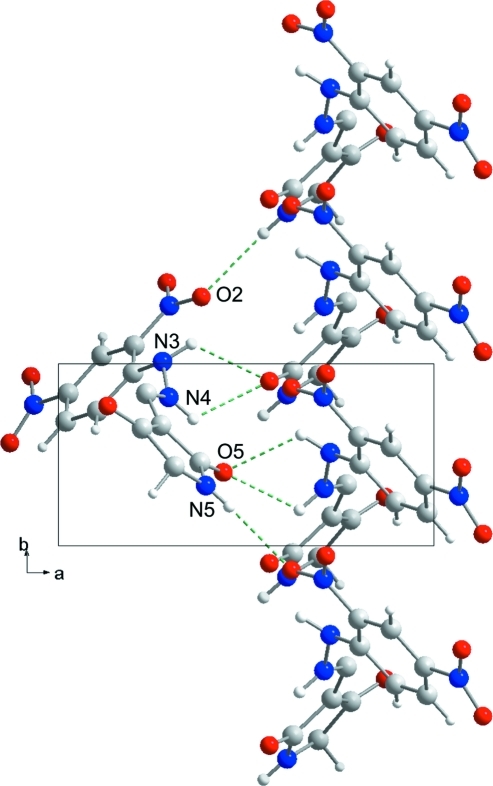
View of the crystal packing of the title compound, projected down c. Infinite one-dimensional chains along the [010] direction are formed via strong hydrogen-bonding interactions (indicated by green dashed lines). The intramolecular hydrogen bonds and the isobutyl groups are omitted for clarity.

## supplementary crystallographic information

### Comment

The title compound is the condensation product of the *Alternaria* spp. mycotoxin tenuazonic acid and 2,4-dinitrophenylhydrazine. It is formed during the derivatization step of a novel HPLC-ESI multistage MS method for tenuazonic acid quantification in cereals (Siegel *et al.*, 2009). While tenuazonic acid itself occurs as a non-crystallizable gum, the crystal structure of its copper salt has previously been reported (Dippenaar *et al.*, 1977). For exemplary crystal structures of other 2,4-dinitrophenylhydrazones see Tameem *et al.*, 2006, Monfared *et al.*, 2007, Valente *et al.*, 2008, Yin *et al.*, 2008. The structure of the title compound is of particular interest, since solubilized tetramic acids and their hydrazones display a variety of tautomeric forms (Yamaguchi *et al.*, 1976*a,b*, Nolte *et al.*, 1980, Gelin *et al.*, 1982, Royles, 1995, Siegel *et al.*, 2009) (see Fig. 1). While the two rotameric groups I—II and III-IV (Fig. 1) may be differentiated using 1*H*-NMR, the tautomeric equilibria which are fast on the NMR timescale can not be characterized like that. Furthermore, although common NMR experiments allow for the differentiation of the two rotameric tautomers, the structural assignment of the predominant species is not possible. The presented crystal structure indicates that a six-membered ring involving an intramolecular hydrogen bond between the O5 and N4 is in fact favoured for this compound. On the basis of the presented crystal structure, it can also be assumed, that the thermodynamically favoured tautomer does not involve a double bond of N3 or N4 and thus is tautomer I (Fig. 1). Six N—H···O hydrogen bonds connect each molecule to four adjacent molecules, which are all screw images and span a length of four unit cells. As depicted in Fig. 3 these interactions result in indefinite chains along the b axis.

### Experimental

The tenuazonic acid natrium salt was supplied by the workgroup of Professor R. Faust (University of Kassel, Germany) by total synthesis from *L*-isoleucine according to a literature procedure (Lebrun *et al.*, 1988). The title compound was synthesized by adding the tenuazonic acid natrium salt (1 eq.) to a 15 m*M* solution of 2,4-dinitrophenylhydrazine in 2 N HCl (2 eq.). After 30 minutes of shaking the precipitate was collected, washed with water, dissolved in ethyl acetate and dried with natrium sulfate. After evaporation of the solvent, a yellow powder was obtained, which was recrystallized from ethanol five times to obtain the title compound in analytical purity. For X-ray analysis yellow crystals of tenuazonic acid 2,4-dinitrophenylhydrazone were grown by solvent evaporation from ethanol at ambient temperature over a period of three weeks.

### Refinement

The hydrogen atoms were located in difference maps but positioned with idealized geometry and refined using the riding model, with N,C—H = 0.93–0.97 Å, and *U*ĩso~(H) = 1.2*U*~eq~(parent atom). Methyl groups (C14, C15, C16) were refined with *U*ĩso~(H) = 1.5*U*~eq~(parent atom).

### Crystal data

**Table:**

C_16_H_19_N_5_O_6_	*F*(000) = 396
*M**_r_* = 377.36	*D*_x_ = 1.480 Mg m^−^^3^
Monoclinic, *P*2_1_	Cu *K*α radiation, λ = 1.54178 Å
Hall symbol: P 2yb	Cell parameters from 25 reflections
*a* = 10.671 (1) Å	θ = 60–69°
*b* = 4.9387 (5) Å	µ = 0.98 mm^−^^1^
*c* = 16.839 (2) Å	*T* = 193 K
β = 107.363 (4)°	Needles, yellow
*V* = 846.98 (15) Å^3^	0.64 × 0.06 × 0.06 mm
*Z* = 2	

### Data collection

**Table:**

Enraf–Nonius CAD-4 diffractometer	3103 reflections with *I* > 2σ(*I*)
Radiation source: fine-focus sealed tube	*R*_int_ = 0.025
graphite	θ_max_ = 73.6°, θ_min_ = 2.8°
ω/2θ scans	*h* = −13→13
Absorption correction: ψ scan (CORINC; Dräger & Gattow, 1971)	*k* = −5→6
*T*_min_ = 0.78, *T*_max_ = 0.94	*l* = −20→20
3890 measured reflections	3 standard reflections every 60 min
3282 independent reflections	intensity decay: 2%

### Refinement

**Table:**

Refinement on *F*^2^	Secondary atom site location: difference Fourier map
Least-squares matrix: full	Hydrogen site location: inferred from neighbouring sites
*R*[*F*^2^ > 2σ(*F*^2^)] = 0.039	H-atom parameters constrained
*wR*(*F*^2^) = 0.111	*w* = 1/[σ^2^(*F*_o_^2^) + (0.0778*P*)^2^ + 0.1634*P*] where *P* = (*F*_o_^2^ + 2*F*_c_^2^)/3
*S* = 0.99	(Δ/σ)_max_ < 0.001
3282 reflections	Δρ_max_ = 0.24 e Å^−^^3^
247 parameters	Δρ_min_ = −0.21 e Å^−^^3^
1 restraint	Absolute structure: (Flack,1983)
Primary atom site location: structure-invariant direct methods	Flack parameter: 0.1 (2)

### Special details

**Table:**

Geometry. All e.s.d.'s (except the e.s.d. in the dihedral angle between two l.s. planes) are estimated using the full covariance matrix. The cell e.s.d.'s are taken into account individually in the estimation of e.s.d.'s in distances, angles and torsion angles; correlations between e.s.d.'s in cell parameters are only used when they are defined by crystal symmetry. An approximate (isotropic) treatment of cell e.s.d.'s is used for estimating e.s.d.'s involving l.s. planes.
Refinement. Refinement of *F*^2^ against ALL reflections. The weighted *R*-factor *wR* and goodness of fit *S* are based on *F*^2^, conventional *R*-factors *R* are based on *F*, with *F* set to zero for negative *F*^2^. The threshold expression of *F*^2^ > σ(*F*^2^) is used only for calculating *R*-factors(gt) *etc*. and is not relevant to the choice of reflections for refinement. *R*-factors based on *F*^2^ are statistically about twice as large as those based on *F*, and *R*- factors based on ALL data will be even larger.

### Fractional atomic coordinates and isotropic or equivalent isotropic displacement parameters (Å^2^)

**Table:**

	*x*	*y*	*z*	*U*_iso_*/*U*_eq_	
O1	0.28853 (17)	1.4445 (4)	0.18665 (10)	0.0394 (4)	
O2	0.38017 (15)	1.3663 (4)	0.31656 (10)	0.0373 (4)	
O3	−0.1297 (2)	0.5644 (5)	0.06973 (11)	0.0559 (5)	
O4	−0.07342 (18)	0.9300 (4)	0.01932 (9)	0.0444 (4)	
O5	0.43857 (13)	0.4033 (3)	0.54411 (8)	0.0277 (3)	
O6	0.13063 (14)	0.7678 (4)	0.66874 (9)	0.0348 (4)	
N1	0.29520 (16)	1.3174 (4)	0.24909 (10)	0.0267 (4)	
N2	−0.06562 (18)	0.7738 (4)	0.07614 (11)	0.0339 (4)	
N3	0.27686 (16)	1.0106 (4)	0.39501 (10)	0.0280 (4)	
H3N	0.3538	1.1087	0.4007	0.034*	
N4	0.29331 (16)	0.8153 (4)	0.45682 (10)	0.0266 (4)	
H4N	0.3584	0.6959	0.4627	0.032*	
N5	0.39181 (16)	0.3303 (4)	0.66688 (9)	0.0262 (4)	
H5N	0.4542	0.2158	0.6856	0.031*	
C1	0.20134 (18)	1.0983 (4)	0.24520 (12)	0.0230 (4)	
C2	0.11472 (18)	1.0434 (4)	0.16609 (11)	0.0254 (4)	
H2	0.1175	1.1462	0.1190	0.030*	
C3	0.02556 (19)	0.8361 (5)	0.15868 (12)	0.0265 (4)	
C4	0.01923 (18)	0.6831 (4)	0.22646 (12)	0.0271 (4)	
H4	−0.0428	0.5404	0.2195	0.032*	
C5	0.10350 (19)	0.7399 (4)	0.30371 (12)	0.0266 (4)	
H5	0.0984	0.6356	0.3500	0.032*	
C6	0.19821 (17)	0.9503 (4)	0.31639 (11)	0.0240 (4)	
C7	0.23382 (17)	0.8356 (4)	0.51605 (11)	0.0231 (4)	
C8	0.27239 (16)	0.6612 (4)	0.58383 (11)	0.0221 (4)	
C9	0.37640 (18)	0.4572 (4)	0.59406 (11)	0.0212 (4)	
C10	0.30819 (18)	0.4349 (4)	0.71460 (11)	0.0242 (4)	
H10	0.2515	0.2860	0.7250	0.029*	
C11	0.22234 (17)	0.6447 (4)	0.65454 (11)	0.0246 (4)	
C12	0.38790 (18)	0.5633 (4)	0.79844 (11)	0.0253 (4)	
H12	0.4282	0.7347	0.7860	0.030*	
C13	0.4990 (3)	0.3758 (6)	0.84642 (14)	0.0444 (6)	
H13A	0.4600	0.2030	0.8572	0.053*	
H13B	0.5552	0.3350	0.8106	0.053*	
C14	0.5849 (3)	0.4865 (7)	0.92845 (14)	0.0501 (7)	
H14A	0.5337	0.4985	0.9680	0.075*	
H14B	0.6165	0.6671	0.9197	0.075*	
H14C	0.6599	0.3656	0.9508	0.075*	
C15	0.2971 (2)	0.6350 (9)	0.84970 (15)	0.0578 (9)	
H15A	0.3448	0.7459	0.8974	0.087*	
H15B	0.2664	0.4686	0.8696	0.087*	
H15C	0.2216	0.7368	0.8152	0.087*	
C16	0.12597 (18)	1.0397 (4)	0.50566 (12)	0.0278 (4)	
H16A	0.0503	0.9865	0.4589	0.042*	
H16B	0.1572	1.2181	0.4945	0.042*	
H16C	0.0999	1.0476	0.5567	0.042*	

### Atomic displacement parameters (Å^2^)

**Table:**

	*U*^11^	*U*^22^	*U*^33^	*U*^12^	*U*^13^	*U*^23^
O1	0.0475 (9)	0.0367 (10)	0.0360 (8)	−0.0111 (7)	0.0159 (7)	0.0053 (7)
O2	0.0333 (7)	0.0348 (9)	0.0376 (7)	−0.0119 (7)	0.0012 (6)	0.0042 (7)
O3	0.0587 (11)	0.0619 (14)	0.0401 (9)	−0.0337 (11)	0.0039 (8)	−0.0078 (9)
O4	0.0529 (9)	0.0479 (11)	0.0256 (7)	−0.0039 (9)	0.0015 (6)	0.0023 (8)
O5	0.0263 (6)	0.0311 (8)	0.0265 (6)	0.0058 (6)	0.0092 (5)	−0.0005 (6)
O6	0.0290 (7)	0.0447 (10)	0.0320 (7)	0.0120 (7)	0.0111 (5)	−0.0006 (7)
N1	0.0266 (8)	0.0226 (9)	0.0316 (8)	−0.0008 (7)	0.0099 (6)	0.0016 (7)
N2	0.0334 (9)	0.0378 (12)	0.0279 (8)	−0.0041 (8)	0.0053 (7)	−0.0050 (8)
N3	0.0256 (7)	0.0300 (10)	0.0246 (8)	−0.0046 (7)	0.0020 (6)	0.0053 (7)
N4	0.0250 (7)	0.0276 (9)	0.0260 (8)	0.0049 (7)	0.0059 (6)	0.0050 (7)
N5	0.0311 (8)	0.0242 (9)	0.0226 (7)	0.0071 (7)	0.0073 (6)	0.0004 (6)
C1	0.0219 (8)	0.0195 (10)	0.0277 (9)	0.0018 (7)	0.0075 (7)	0.0015 (7)
C2	0.0262 (9)	0.0244 (10)	0.0257 (9)	0.0029 (8)	0.0082 (7)	0.0012 (8)
C3	0.0236 (8)	0.0290 (11)	0.0257 (9)	0.0016 (8)	0.0053 (7)	−0.0028 (8)
C4	0.0239 (9)	0.0249 (11)	0.0336 (9)	−0.0028 (8)	0.0104 (7)	−0.0028 (8)
C5	0.0280 (9)	0.0253 (11)	0.0276 (9)	−0.0003 (8)	0.0099 (7)	0.0031 (8)
C6	0.0210 (8)	0.0254 (11)	0.0254 (8)	0.0021 (7)	0.0064 (7)	−0.0011 (8)
C7	0.0204 (8)	0.0213 (9)	0.0239 (8)	−0.0048 (7)	0.0009 (6)	−0.0041 (7)
C8	0.0200 (8)	0.0209 (10)	0.0228 (8)	0.0004 (7)	0.0025 (6)	−0.0032 (7)
C9	0.0218 (8)	0.0175 (10)	0.0221 (8)	−0.0014 (7)	0.0030 (6)	−0.0026 (7)
C10	0.0254 (8)	0.0240 (10)	0.0237 (8)	−0.0011 (8)	0.0083 (7)	−0.0014 (8)
C11	0.0206 (8)	0.0266 (11)	0.0244 (8)	−0.0004 (8)	0.0031 (6)	−0.0037 (8)
C12	0.0274 (9)	0.0266 (11)	0.0209 (8)	0.0008 (8)	0.0056 (7)	−0.0016 (7)
C13	0.0492 (13)	0.0449 (16)	0.0314 (10)	0.0177 (12)	0.0005 (9)	0.0001 (10)
C14	0.0420 (13)	0.071 (2)	0.0305 (11)	0.0011 (13)	−0.0001 (9)	0.0054 (12)
C15	0.0391 (12)	0.102 (3)	0.0311 (11)	0.0168 (15)	0.0088 (9)	−0.0188 (14)
C16	0.0229 (8)	0.0242 (11)	0.0330 (10)	0.0017 (8)	0.0032 (7)	0.0003 (8)

### Geometric parameters (Å, °)

**Table:**

O1—N1	1.208 (2)	C5—H5	0.9500
O2—N1	1.248 (2)	C7—C8	1.391 (3)
O3—N2	1.227 (3)	C7—C16	1.500 (3)
O4—N2	1.212 (3)	C8—C11	1.445 (3)
O5—C9	1.246 (2)	C8—C9	1.470 (3)
O6—C11	1.235 (2)	C10—C11	1.544 (3)
N1—C1	1.463 (3)	C10—C12	1.550 (2)
N2—C3	1.472 (2)	C10—H10	1.0000
N3—C6	1.372 (2)	C12—C15	1.519 (3)
N3—N4	1.391 (2)	C12—C13	1.531 (3)
N3—H3N	0.9328	C12—H12	1.0000
N4—C7	1.336 (2)	C13—C14	1.514 (3)
N4—H4N	0.8942	C13—H13A	0.9900
N5—C9	1.343 (2)	C13—H13B	0.9900
N5—C10	1.462 (2)	C14—H14A	0.9800
N5—H5N	0.8585	C14—H14B	0.9800
C1—C2	1.403 (3)	C14—H14C	0.9800
C1—C6	1.413 (3)	C15—H15A	0.9800
C2—C3	1.378 (3)	C15—H15B	0.9800
C2—H2	0.9500	C15—H15C	0.9800
C3—C4	1.387 (3)	C16—H16A	0.9800
C4—C5	1.372 (3)	C16—H16B	0.9800
C4—H4	0.9500	C16—H16C	0.9800
C5—C6	1.421 (3)		
			
O1—N1—O2	121.99 (18)	N5—C9—C8	108.01 (16)
O1—N1—C1	118.93 (16)	N5—C10—C11	102.55 (14)
O2—N1—C1	119.08 (16)	N5—C10—C12	112.72 (16)
O4—N2—O3	124.19 (18)	C11—C10—C12	112.29 (17)
O4—N2—C3	118.86 (19)	N5—C10—H10	109.7
O3—N2—C3	116.95 (19)	C11—C10—H10	109.7
C6—N3—N4	118.40 (17)	C12—C10—H10	109.7
C6—N3—H3N	118.5	O6—C11—C8	129.87 (19)
N4—N3—H3N	111.9	O6—C11—C10	123.60 (17)
C7—N4—N3	121.63 (17)	C8—C11—C10	106.53 (16)
C7—N4—H4N	119.6	C15—C12—C13	111.46 (19)
N3—N4—H4N	117.4	C15—C12—C10	110.08 (17)
C9—N5—C10	114.12 (17)	C13—C12—C10	111.18 (18)
C9—N5—H5N	120.9	C15—C12—H12	108.0
C10—N5—H5N	124.4	C13—C12—H12	108.0
C2—C1—C6	122.02 (18)	C10—C12—H12	108.0
C2—C1—N1	115.71 (16)	C14—C13—C12	115.2 (2)
C6—C1—N1	122.27 (16)	C14—C13—H13A	108.5
C3—C2—C1	118.11 (18)	C12—C13—H13A	108.5
C3—C2—H2	120.9	C14—C13—H13B	108.5
C1—C2—H2	120.9	C12—C13—H13B	108.5
C2—C3—C4	122.15 (17)	H13A—C13—H13B	107.5
C2—C3—N2	119.00 (18)	C13—C14—H14A	109.5
C4—C3—N2	118.85 (19)	C13—C14—H14B	109.5
C5—C4—C3	119.32 (19)	H14A—C14—H14B	109.5
C5—C4—H4	120.3	C13—C14—H14C	109.5
C3—C4—H4	120.3	H14A—C14—H14C	109.5
C4—C5—C6	121.87 (18)	H14B—C14—H14C	109.5
C4—C5—H5	119.1	C12—C15—H15A	109.5
C6—C5—H5	119.1	C12—C15—H15B	109.5
N3—C6—C1	122.96 (18)	H15A—C15—H15B	109.5
N3—C6—C5	120.45 (17)	C12—C15—H15C	109.5
C1—C6—C5	116.52 (17)	H15A—C15—H15C	109.5
N4—C7—C8	118.42 (17)	H15B—C15—H15C	109.5
N4—C7—C16	118.82 (17)	C7—C16—H16A	109.5
C8—C7—C16	122.73 (17)	C7—C16—H16B	109.5
C7—C8—C11	128.27 (17)	H16A—C16—H16B	109.5
C7—C8—C9	123.28 (16)	C7—C16—H16C	109.5
C11—C8—C9	108.45 (16)	H16A—C16—H16C	109.5
O5—C9—N5	124.91 (18)	H16B—C16—H16C	109.5
O5—C9—C8	127.06 (17)		
			
C6—N3—N4—C7	−105.2 (2)	N4—C7—C8—C11	−179.71 (18)
O1—N1—C1—C2	3.8 (3)	C16—C7—C8—C11	−1.4 (3)
O2—N1—C1—C2	−175.79 (18)	N4—C7—C8—C9	0.7 (3)
O1—N1—C1—C6	−176.46 (19)	C16—C7—C8—C9	178.93 (17)
O2—N1—C1—C6	4.0 (3)	C10—N5—C9—O5	179.12 (18)
C6—C1—C2—C3	−0.8 (3)	C10—N5—C9—C8	−2.4 (2)
N1—C1—C2—C3	179.01 (17)	C7—C8—C9—O5	−3.5 (3)
C1—C2—C3—C4	0.1 (3)	C11—C8—C9—O5	176.79 (19)
C1—C2—C3—N2	−179.57 (18)	C7—C8—C9—N5	178.00 (18)
O4—N2—C3—C2	−10.8 (3)	C11—C8—C9—N5	−1.7 (2)
O3—N2—C3—C2	169.4 (2)	C9—N5—C10—C11	5.1 (2)
O4—N2—C3—C4	169.5 (2)	C9—N5—C10—C12	−115.88 (19)
O3—N2—C3—C4	−10.3 (3)	C7—C8—C11—O6	4.6 (4)
C2—C3—C4—C5	0.5 (3)	C9—C8—C11—O6	−175.7 (2)
N2—C3—C4—C5	−179.82 (18)	C7—C8—C11—C10	−174.97 (18)
C3—C4—C5—C6	−0.5 (3)	C9—C8—C11—C10	4.7 (2)
N4—N3—C6—C1	−162.77 (18)	N5—C10—C11—O6	174.63 (19)
N4—N3—C6—C5	20.5 (3)	C12—C10—C11—O6	−64.1 (2)
C2—C1—C6—N3	−176.16 (18)	N5—C10—C11—C8	−5.7 (2)
N1—C1—C6—N3	4.1 (3)	C12—C10—C11—C8	115.53 (17)
C2—C1—C6—C5	0.7 (3)	N5—C10—C12—C15	−172.8 (2)
N1—C1—C6—C5	−179.02 (17)	C11—C10—C12—C15	72.0 (3)
C4—C5—C6—N3	176.88 (19)	N5—C10—C12—C13	−48.8 (2)
C4—C5—C6—C1	−0.1 (3)	C11—C10—C12—C13	−164.01 (19)
N3—N4—C7—C8	−168.70 (16)	C15—C12—C13—C14	−58.2 (3)
N3—N4—C7—C16	13.0 (3)	C10—C12—C13—C14	178.5 (2)

### Hydrogen-bond geometry (Å, °)

**Table:**

*D*—H···*A*	*D*—H	H···*A*	*D*···*A*	*D*—H···*A*
N3—H3N···O2	0.93	1.99	2.630 (2)	125
N4—H4N···O5	0.89	2.00	2.710 (2)	135
N3—H3N···O5^i^	0.93	2.36	2.949 (2)	121
N4—H4N···O5^i^	0.89	2.43	2.898 (2)	113
N5—H5N···O2^ii^	0.86	2.48	3.293 (2)	159

Symmetry codes: (i) −*x*+1, *y*+1/2, −*z*+1; (ii) −*x*+1, *y*−3/2, −*z*+1.
